# Chronic kidney diseases and inflammation research: a bibliometric analysis

**DOI:** 10.3389/fmed.2024.1388665

**Published:** 2024-09-20

**Authors:** Heyong Wang, Yang Chen, Yujuan Gou, Dianxing Yang, Lanyue Xiong

**Affiliations:** ^1^Department of Nephrology, Sichuan Integrative Medicine Hospital, Chengdu, China; ^2^School of Basic Medical Sciences, Chengdu University of Traditional Chinese Medicine, Chengdu, China; ^3^Department of Gastroenterology, Chengdu Eighth People's Hospital, Chengdu, China; ^4^Department of Cardiovascular, Chengdu First People's Hospital, Chengdu, China

**Keywords:** chronic kidney diseases, inflammation, bibliometrics, VOSviewer, CiteSpace, oxidative stress

## Abstract

**Background:**

Chronic kidney diseases (CKD) is a severe public health problem. This study aimed to explore the field of inflammation-related research in CKD from a bibliometric perspective.

**Methods:**

Relevant literature published between 2004 and 2023 were searched from the Web of Science database. The bibliometric analysis were performed to summarize countries, institutions, authors, journals and keywords using VOSviewer and CiteSpace.

**Results:**

A total of 9,287 publications on CKD and inflammation were included. Publications were mainly from the United States, China, Italy, Germany, and Japan. The findings revealed that the United States had the highest number of publications in this field, followed by China. There is strong collaboration between the two countries. The most productive institutions included the University of California system and the US Department of Veterans Affairs. Research hotspots primarily focused on inflammation mechanisms, biomarkers, and interventions.

**Conclusion:**

This study revealed the basic knowledge structure and provided a comprehensive insight into the research field of CKD and inflammation through bibliometric methods. Future studies should focus on early diagnosis, prevention, and treatment strategies of CKD, and explore more inflammation associated biomarkers and therapeutic targets for CKD.

## 1 Introduction

Chronic kidney disease (CKD) is defined as the presence of structural and functional renal dysfunction for at least 3 months and characterized by reduced glomerular filtration rate, albuminuria, renal morphological changes, tubular dysfunction, or a history of kidney transplantation (1). As a significant and growing global public health problem, CKD affects 800 million people worldwide (2), and causes millions of death every year (3), which ranked 16th leading cause of death worldwide (4) Liyanage et al. conducted a study on end-stage kidney disease (ESKD) renal replacement therapy in 123 countries. The findings revealed that in 2010, 2.618 million individuals underwent renal replacement therapy, out of which 2.05 million received dialysis. The prevalence of ESKD treatment exhibited significant regional variation, with 80 cases per million people in Africa and 1840 cases per million people in North America (5). The pathophysiology of CKD manifests as progressive renal damage, which may leads to the progression of renal dysfunction and may eventually progress to end-stage renal disease (ESRD) (6). The economic burden caused by CKD is increasing due to the aging population and prevalence of comorbid diseases (7).

In recent years, the role of inflammation in the pathogenesis of CKD has received extensive attention. One of the key features underlying the progression of CKD is the persistent and systemic inflammation observed in these patients (8, 9). Inflammation plays a crucial role in the initiation and progression of CKD and contributes to the development of various complications, such as cardiovascular diseases (10), malnutrition (11), anemia (12), and renal osteopathy (13). Microinflammatory state is an important risk factor for mortality in chronic kidney disease (14). Therefore, certain inflammatory biomarkers have been reported to be used as possible predictors and therapeutic targets for CKD (15).

Despite the rapidly growth of published literatures on the relationship of inflammation and CKD, there has been limited effort to systematically analyze and map the research landscape in the field. A comprehensive understanding of the current state of research, key players, and emerging trends is essential for guiding future research directions and identifying knowledge gaps.

Bibliometric analysis is a powerful tool for analyzing scientific literatures and visualizing research landscapes. It provides insights into influential countries, institutions, authors, and journals. It also helps to identify research hotspots and emerging trends (16). For instance, a bibliometric analysis reported the worldwide research patterns and focal points concerning autophagy and kidney disease between 2000 and 2022 (17), while another bibliometric analysis has been conducted on the topic of schizophrenia and inflammation studies (18). Nevertheless, as of now, no bibliometric analysis has been published regarding the subject of inflammation and CKD. In order to fill this research gap, we conducted this bibliometric analysis study to explore the field of inflammation-related research in CKD.

## 2 Methods

### 2.1 Data acquisition and search strategy

The literatures related to the role of inflammation in CKD from January 1st, 2004 to December 31, 2023 were searched in the Web of Science Core Collection (WOSCC) database on January 20, 2024.The search formula was as follows: (((((((((((((((((((TS = (Renal Insufficiency, Chronic)) OR TS = (Chronic Renal Insufficiencies)) OR TS = (Renal Insufficiencies, Chronic)) OR TS = (Chronic Renal Insufficiency)) OR TS = (Kidney Insufficiency, Chronic)) OR TS = (Chronic Kidney Insufficiency)) OR TS = (Chronic Kidney Insufficiencies)) OR TS = (Kidney Insufficiencies, Chronic)) OR TS = (Chronic Kidney Diseases)) OR TS = (Chronic Kidney Disease)) OR TS = (Disease, Chronic Kidney)) OR TS = (Diseases, Chronic Kidney)) OR TS = (Kidney Disease, Chronic)) OR TS = (Kidney Diseases, Chronic)) OR TS = (Chronic Renal Diseases)) OR TS = (Chronic Renal Disease)) OR TS = (Disease, Chronic Renal)) OR TS = (Diseases, Chronic Renal)) OR TS = (Renal Disease, Chronic)) OR TS = (Renal Diseases, Chronic) AND ((((TS = (inflammatory) OR TS = (inflammation biomarkers) OR TS = (oxidative stress biomarkers)OR TS = (endothelial biomarkers). Literature types including regular and review articles were retrieved without publication language restrictions. The relevant articles were exported and stored in the form of plain.txt (including full records and cited references) for further analyses.

### 2.2 Selection criteria

(1) The full-text articles were related to the role of inflammation in CKD; (2) Both the research and review articles were written in English; (3) The articles were published between January 1st, 2004 and December 31, 2023.

### 2.3 Exclusion criteria

(1) The subjects of the articles were not related to the role of inflammation in CKD; (2) Publications are conference abstracts, news, briefings, etc.

### 2.4 Data analysis

GraphPad Prism v8.0.2 was utilized in this study to analyze and visualize the publishing trend and proportion of annual and national publications (19). In addition, CtieSpace [6.2.4r (64 bit) advanced version] and VOSviewer (version 1.6.18) were used to analyze and visualize the map of scientific knowledge. VOSviewer is a free Java-based software developed by Waltman et al. in 2009. It analyzes and presents extensive literature data in the form of maps (20). Professor Chen Chaomei developed CiteSpace software to visually demonstrate the research findings in a specific field (21). This software allows users to analyze new concepts, assess current technologies, and gain insights into the knowledge field, research frontiers, trends, and future research directions.

## 3 Results

### 3.1 Literature search results

**Figure 1:**
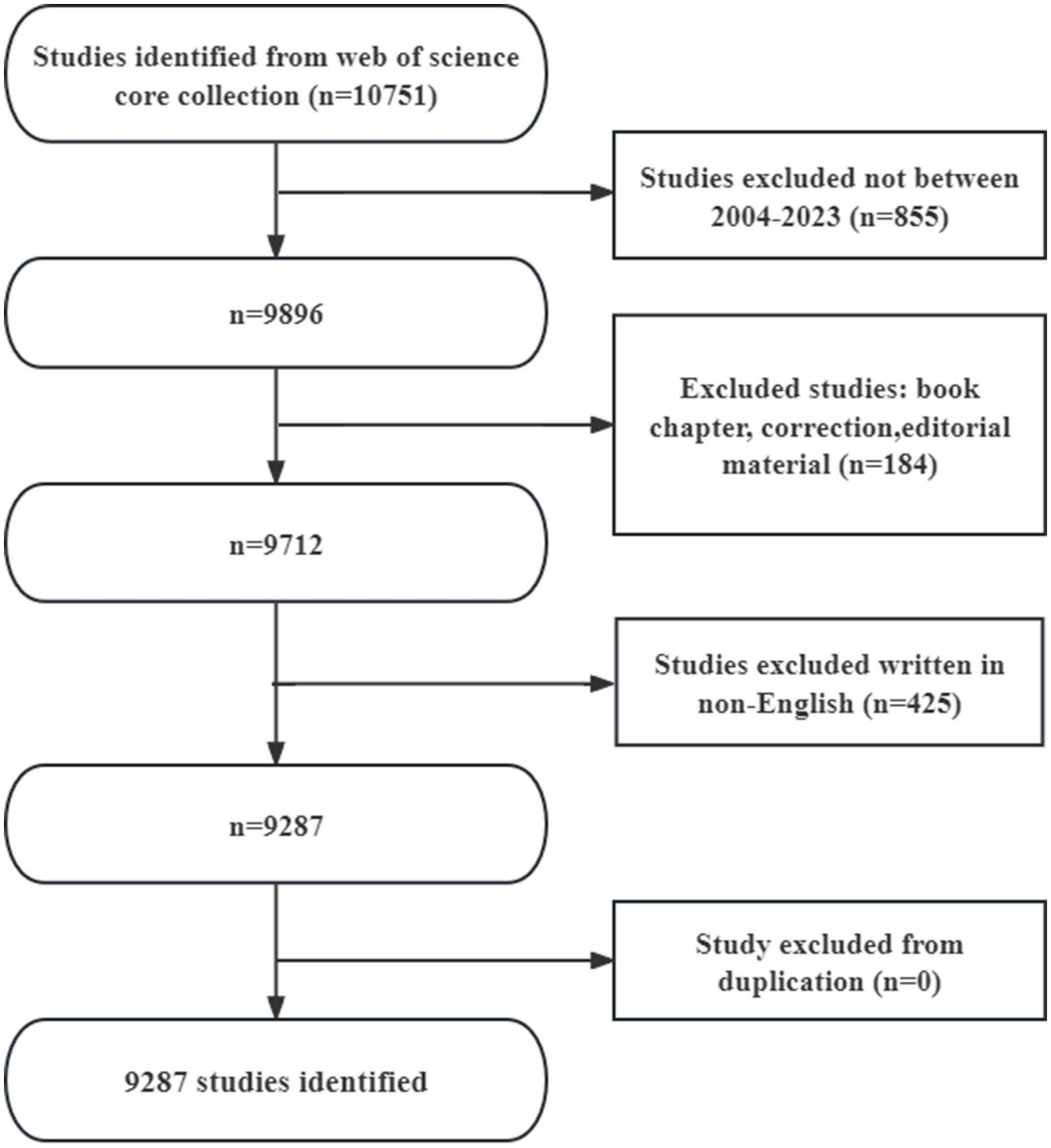
Flow chart of the literature search.

As the literature search process shown in Figure 1, a total of 9,287 articles were enrolled, including 6,873 researches (84.96%) and 2,414 reviews (15.04%). The literatures covers 126 countries and regions, 7,354 institutions and 42,728 authors.

### 3.2 Analysis of publications

**Figure 2:**
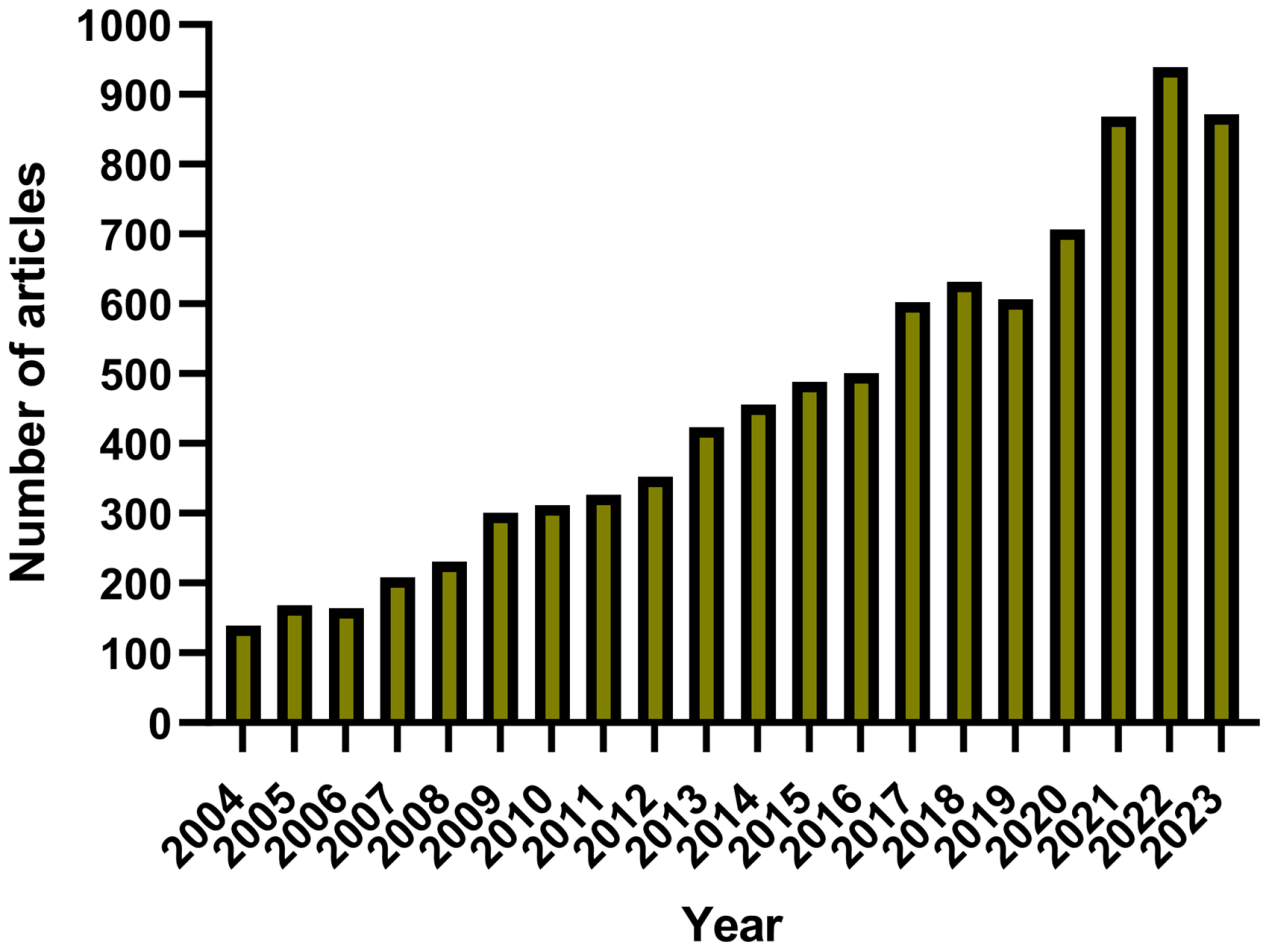
Chart of annual publication volume.

The number of annual publications has increased slowly since 2004 (Figure 2). We divided the process into three stages: 1. from 2004 to 2006, as the annual number of published papers was less than 200, the growth was slow, indicating that this field has not received much attention; 2. from 2007 to 2012, as the number of published papers gradually increased, it indicates that this field has been gradually noticed by the researchers; 3. after 2013, the number of papers published in this field surged and peaked in 2022, indicating that this field has received widespread attention.

### 3.3 Analysis of the most productive countries

**Figure 3:**
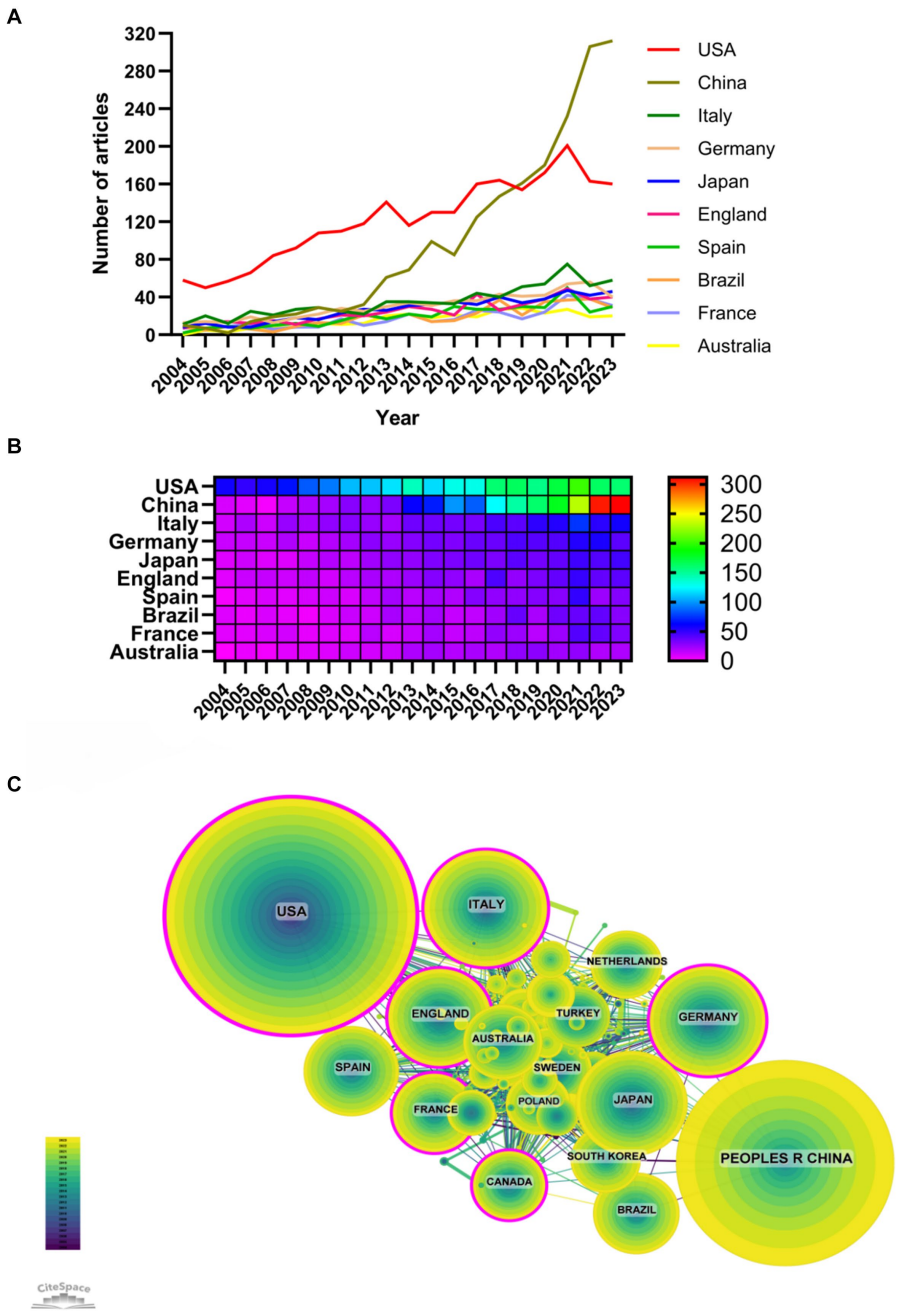
The most productive countries (**A**, line chart of national publications; **B**, heat map of national publications; **C**, national cooperation network).

The role of inflammation in CKD has been studied in 126 countries and regions. Figures 3A,B showed the number of annual publications by the top 10 countries in the past decade. The top five countries are the United States, China, Italy, Germany, and Japan. The number of papers published in the United States accounted for 26.22% of the total, far more than other countries.

Among the top 10 countries/regions in terms of publication volume, the United States has 134,434 citations (Table 1), surpassing all other countries/regions. Its paper citation/publication ratio (55.21) ranks first among all countries/regions, indicating that the quality of its published papers is generally high. China ranks second in terms of publication volume (1937 articles) and second in terms of citation frequency (41290), with a lower citation/publication ratio (21.32), indicating a lower quality of its published articles. As the cooperation network shown in Figure 3C, there is a close cooperation between China and the United States with the highest production volume. The United States also has close cooperation with other countries such as Italy, England, and France, while China has cooperation with countries such as Japan, South Korea, and Germany. The United States has the largest number of publications and the highest citation frequency, which is in a leading position in this field. In recent years, the number of publications in countries such as China and Italy also has rapidly increased, which may be related to cooperation with the United States.

**Table 1:** Top 10 countries with the most publications.

Rank	Country/region	Article counts	centrality	Percentage (%)	Citation	Citation per publication
1	USA	2,435	0.25	26.22	134,434	55.21
2	China	1937	0.00	20.86	41,290	21.32
3	Italy	704	0.11	7.58	28,784	40.89
4	Germany	608	0.12	6.55	30,027	49.39
5	Japan	537	0.07	5.78	17,196	32.02
6	England	498	0.20	5.36	27,020	54.26
7	Spain	408	0.08	4.39	20,237	49.60
8	Brazil	374	0.05	4.03	9,904	26.48
9	France	351	0.15	3.78	16,465	46.91
10	Australia	309	0.05	3.33	16,039	51.91

### 3.4 Analysis of the most productive institutions

**Figure 4:**
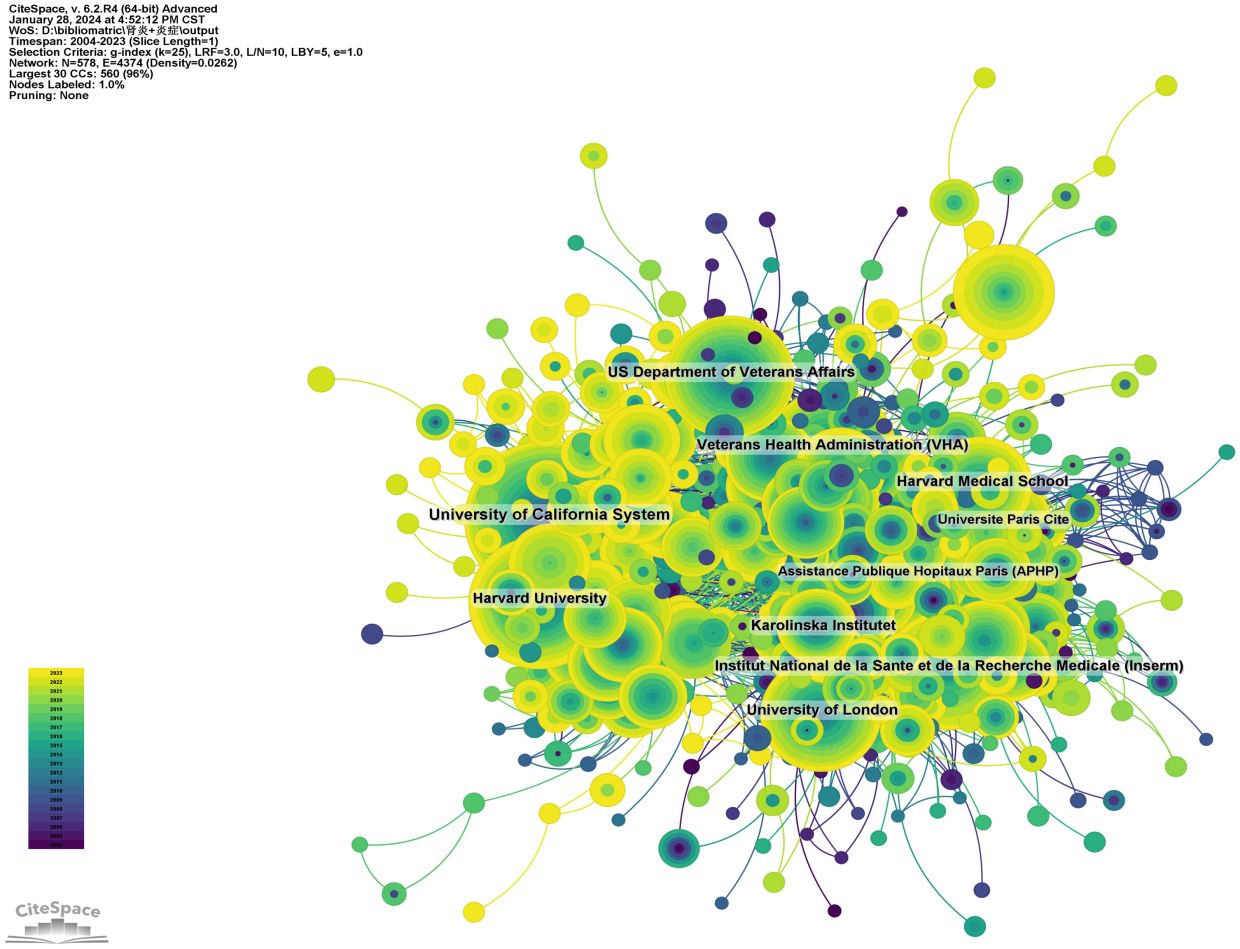
Institutional cooperation network diagram.

7,354 institutions have contributed to researches on the role of inflammation in CKD. Among the top 10 institutions in terms of the publication numbers, 5 are from the United States, 3 from France, 1 from Sweden, and 1 from England (Table 2; Figure 4). The University of California system published the most articles (319 papers, 21,214 citations, 66.50 citations per paper). The US Department of Veterans Affairs (221 papers, 11,335 citations, 51.29 times/article) ranked second, and the Veterans Health Administration (VHA; 212 papers, 10,719 citations, 50.56 times/article) ranked third. The institutional cooperation network showed that institutions were inclined to cooperate with their own domestic units. Therefore, it should be encouraged to strengthen cooperation between domestic and foreign institutions and break academic barriers.

**Table 2:** Top 10 institutions with the most publications.

Rank	Institution	Country	Number of studies	Total citations	Average citation
1	University of California System	USA	319	21,214	66.50
2	US Department of Veterans Affairs	USA	221	11,335	51.29
3	Veterans Health Administration (VHA)	USA	212	10,719	50.56
4	Harvard University	USA	211	11,851	56.17
5	Karolinska Institutet	Sweden	204	10,400	50.98
6	Institut National de la Sante et de la Recherche Medicale (Inserm)	France	202	10,686	52.90
7	University of London	England	186	11,824	63.57
8	Harvard Medical School	USA	149	7,789	52.28
9	Assistance Publique Hopitaux Paris (APHP)	France	136	7,507	55.20
10	Universite Paris Cite	France	125	6,766	54.13

### 3.5 Analysis of the higher-impact journals

**Figure 5:**
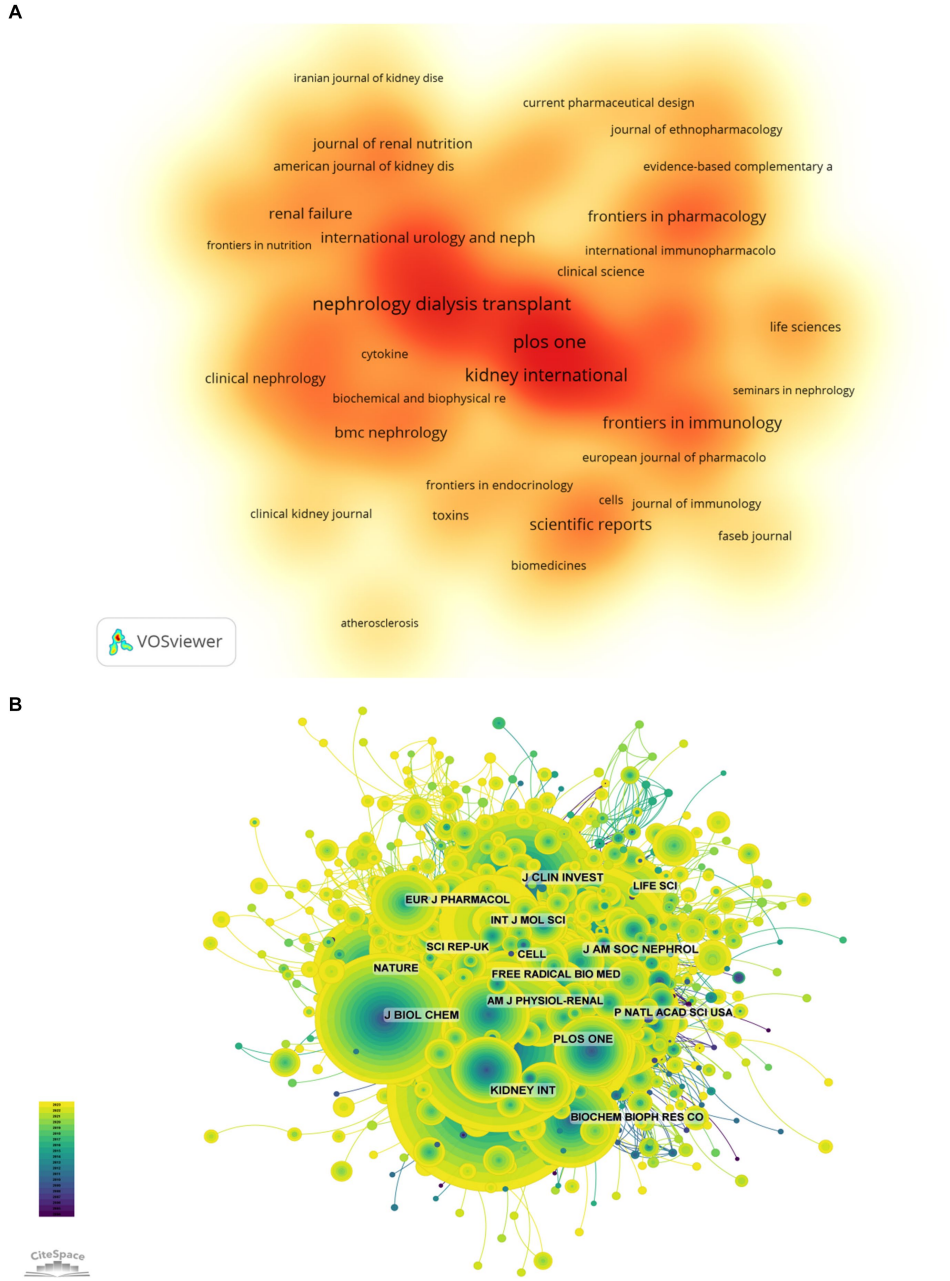
The higher-impact journals (**A**, journal publication density map; **B**, journal citation network diagram).

The top 10 journals with the greatest number of publications and most citations were summarized in the Table 3 and Figure 5A. Nephrology Diagnosis Translation (216 articles, 2.33%) was the most published journal in this field, followed by PLOS One (197 articles, 2.12%), IEEE access (175 articles, 1.88%), Kidney International (171 articles, 1.84%), and International Journal of Molecular Sciences (131 articles, 1.41%). Among the top 10 journals with the most production, the impact factor (IF) of Kidney International is the highest of 19.6. All journals were classified into Q1 or Q2.

**Table 3:** Top 10 journals with the most publications.

Rank	Journal	Article counts	Percentage(9287)	IF	JCR-c
1	Nephrol Dial Transpl	216	2.33	6.1	Q1
2	Plos One	197	2.12	3.7	Q2
3	Kidney Int	175	1.88	19.6	Q1
4	Int J Mol Sci	171	1.84	5.6	Q1
5	Am J Physiol-Renal	131	1.41	4.2	Q1
6	Sci Rep	112	1.21	4.6	Q2
7	Front Immunol	106	1.14	7.3	Q1
8	J Am Soc Nephrol	105	1.13	13.6	Q1
9	Ren Fail	91	0.98	13.6	Q1
10	Front Pharmacol	90	0.97	5.6	Q1

The influence of a journal is determined by its frequency of joint citations, which indicates whether a journal has a significant impact on the scientific community. As shown in Figure 5B and Table 4, the most frequently cited journals are Kidney International (5,774 times), followed by J Am SOC Nephrol (5,201 times) and Nephrol Dial Transpl (4,496 times). Among the top 10 journals with the most joint citations, Lancet has been cited 263 times with the highest IF among the top 10 journals (168.9). All journals were classified into Q1 or Q2.

**Table 4:** Top 10 most co-cited journals.

Rank	Cited journal	Co-citation	IF(2020)	JCR-c
1	Kidney Int	5,774	19.6	Q1
2	J Am Soc Nephrol	5,201	13.6	Q1
3	Nephrol Dial Transpl	4,496	6.1	Q1
4	New Engl J Med	4,117	158.5	Q2
5	Am J Kidney Dis	3,706	13.2	Q1
6	Plos One	3,700	3.7	Q2
7	J Clin Invest	3,403	15.9	Q1
8	Lancet	3,366	168.9	Q1
9	Circulation	2,844	37.8	Q1
10	J Biol Chem	2,734	4.8	Q2

### 3.6 Analysis of the most influential authors

**Figure 6:**
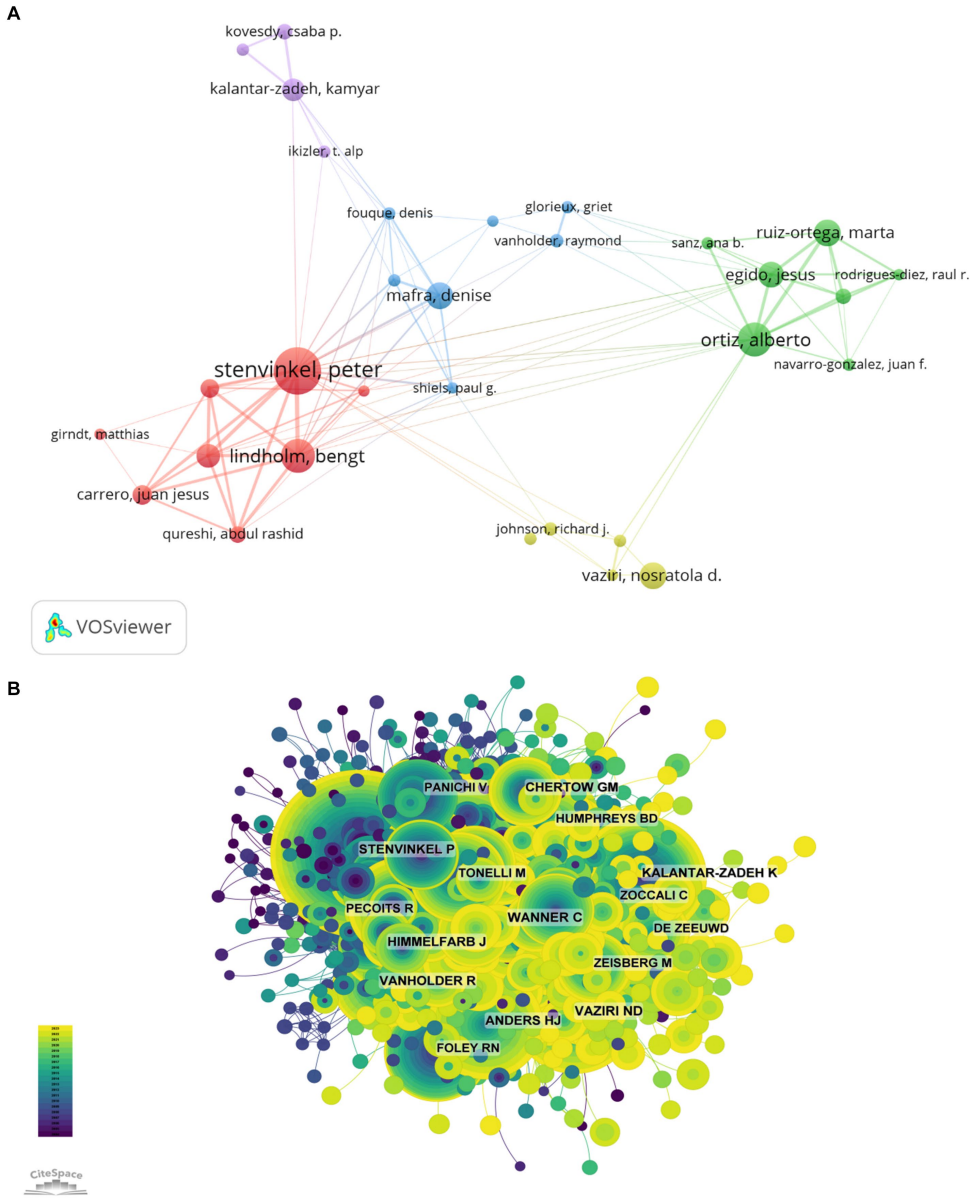
The most influential authors (**A**, author collaboration network diagram; **B**, author citation network diagram).

Among all the authors who have published the relevant literatures on the role of inflammation in CKD, the top 10 authors who have published the most papers were listed in Table 5. The articles published by the top 10 authors were 478, accounting for 5.15% of all publications in this field. Stenvinkel Peter was the most productive, having published 87 research papers, followed by Bengt Lindholm (59), Ortiz Alberto (59), Mafra Denise (44). Further analysis shows that four of the top 10 authors are from Sweden, 3 from Spain, 2 from the United States and 1 from Brazil. The network between authors was visualized with CiteSpace (Figure 6A).

**Table 5:** Top 10 authors with the most publications.

Rank	Author	Count	Location	Rank	Co-cited author	Citation
1	Stenvinkel Peter et al.	87	Sweden	1	Sudarshan P	855
2	Lindholm Bengt et al.	59	Sweden	2	Levey AS	751
3	Ortiz Albertoy et al.	59	Spain	3	Colleen R Kelly	467
4	Mafra Denise et al.	44	Brazil	4	Noortje ND	397
5	Ruiz Marta	43	Spain	5	Anders HJ	381
6	Vaziri Nosratolad et al.	43	USA	6	Ridker PM	378
7	Egido Jesus et al.	41	Spain	7	Go AS	368
8	Barany Peter et al.	37	Sweden	8	Zoccali C	347
9	Kalantar Z Kamyar et al.	35	USA	9	Wang Y	338
10	Carrero J Jesus et al.	30	Sweden	10	Levin A	330

The top 10 most co-cited and most cited authors were summarized in Figure 6B and Table 5, respectively. A total of 132 authors were cited for more than 50 times, indicating their high reputation and influence of their researches. The most co-cited authors were showed with the largest nodes, including LIU Y (73 citations), WANG Y (64 citations), and KENNEDY J (63 citations).

### 3.7 Analysis of research hotspots

#### 3.7.1 The most highly cited article

**Figure 7:**
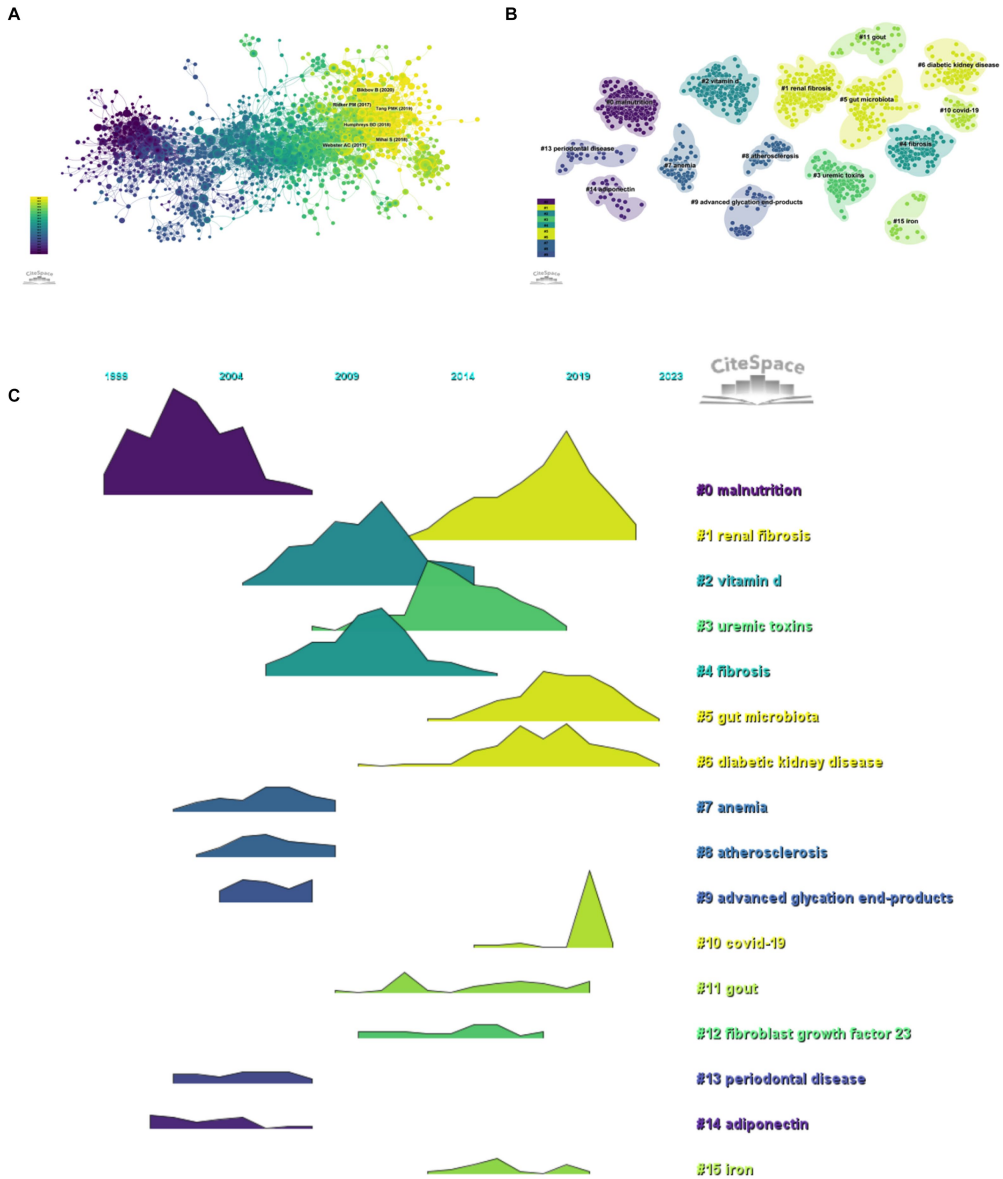
Research hotspots (**A**, citation literature network diagram; **B**, citation literature clustering diagram; **C**, citation literature volcano diagram).

Taking 1 year as the time slice, the cited references network has 1,635 nodes and 7,059 links ranging from 2004 to 2023 (Figure 7A). According to the top 10 articles with the highest number of co citations (Table 6), the article entitled “Chronic kidney disease” in Lancet (IF = 168.9) is the most frequently cited, with Webster, Angela C. as the first authors of this article (22). This article focused on the definition, etiology, clinical manifestations, diagnosis, complications, prognosis, quality of life, intervention measures and service inequality of CKD.

**Table 6:** Top 10 most cited articles.

Rank	Title	Journal IF(2021)	author(s)	Total citations
1	Chronic kidney disease	Lancet (IF = 168.9)	Webster AC	107
2	Global, regional, and national burden of chronic kidney disease, 1990–2017: a systematic analysis for the Global Burden of Disease Study 2017	Lancet (IF = 168.9)	Bikbov B	101
3	Inflammation-Related Mechanisms in Chronic Kidney Disease Prediction, Progression, and Outcome	Journal of Immunology Research (IF = 4.1)	Mihai S	80
4	Antiinflammatory Therapy with Canakinumab for Atherosclerotic Disease	New England Journal of Medicine (IF = 158.5)	Ridker PM	77
5	Mechanisms of Renal Fibrosis	Annual Review of Physiology (IF = 18.2)	Humphreys BD	66
6	Macrophages: versatile players in renal inflammation and fibrosis	Nature Reviews Nephrology (IF = 41.5)	Tang PMK	66
7	TGF-β: the master regulator of fibrosis	Nature Reviews Nephrology (IF = 41.5)	Meng XM	64
8	Global Prevalence of Chronic Kidney Disease - A Systematic Review and Meta-Analysis	Plos One (IF = 3.7)	Hill NR	64
9	Chronic kidney disease and the risks of death, cardiovascular events, and hospitalization	New England Journal of Medicine (IF = 158.5)	Go AS	55
10	Renal tubule injury: a driving force toward chronic kidney disease	Kidney International (IF = 19.6)	Liu BC	50

#### 3.7.2 Analysis of references with citation burst

The co-citation reference clustering and temporal clustering analysis were performed (Figures 7B,C). We found that malnutrition (cluster0) and adiponectin (cluster14) were early research hotspots. Vitamin D (cluster2), uremic toxins (cluster3), fibrosis (cluster4), anemia (cluster7), atherosclerosis (cluster8), advanced glycation end products (cluster9), and periodontal disease (cluster13) were the research hotspots in the medium term. Renal fibrosis (cluster1), gut microbiota (cluster5), diabetic kidney disease (cluster6), COVID-19 (cluster10), gout (cluster11), fibroblast growth factor 23 (cluster12), and iron (cluster15) were hot topics and trends in this field of recent years.

#### 3.7.3 Analysis of the most frequently used keywords

**Figure 8:**
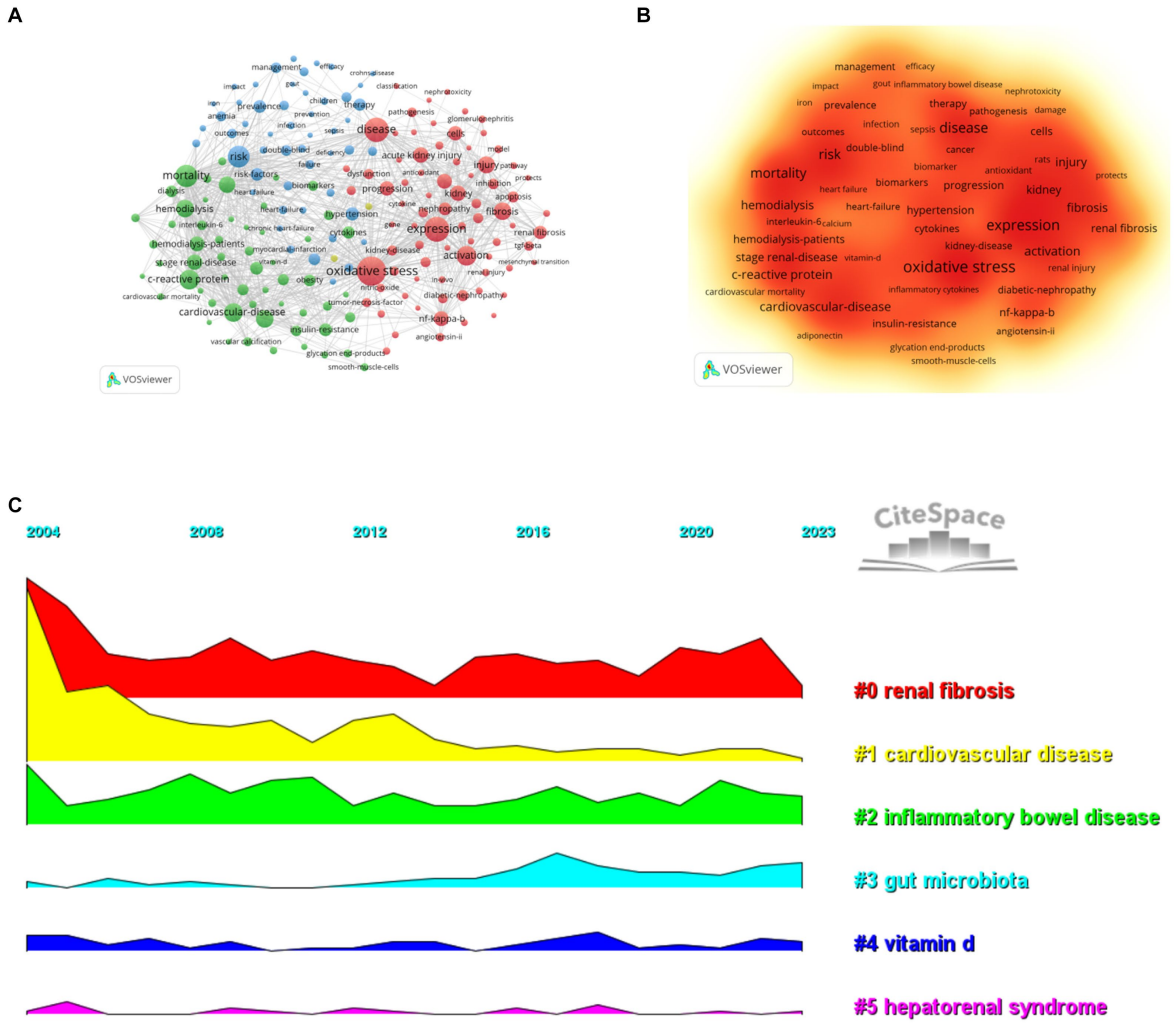
Research hotspots (**A**, high-frequency keyword network diagram; **B**, keyword density map; **C**, keyword clustering volcano diagram).

**Figure 9:**
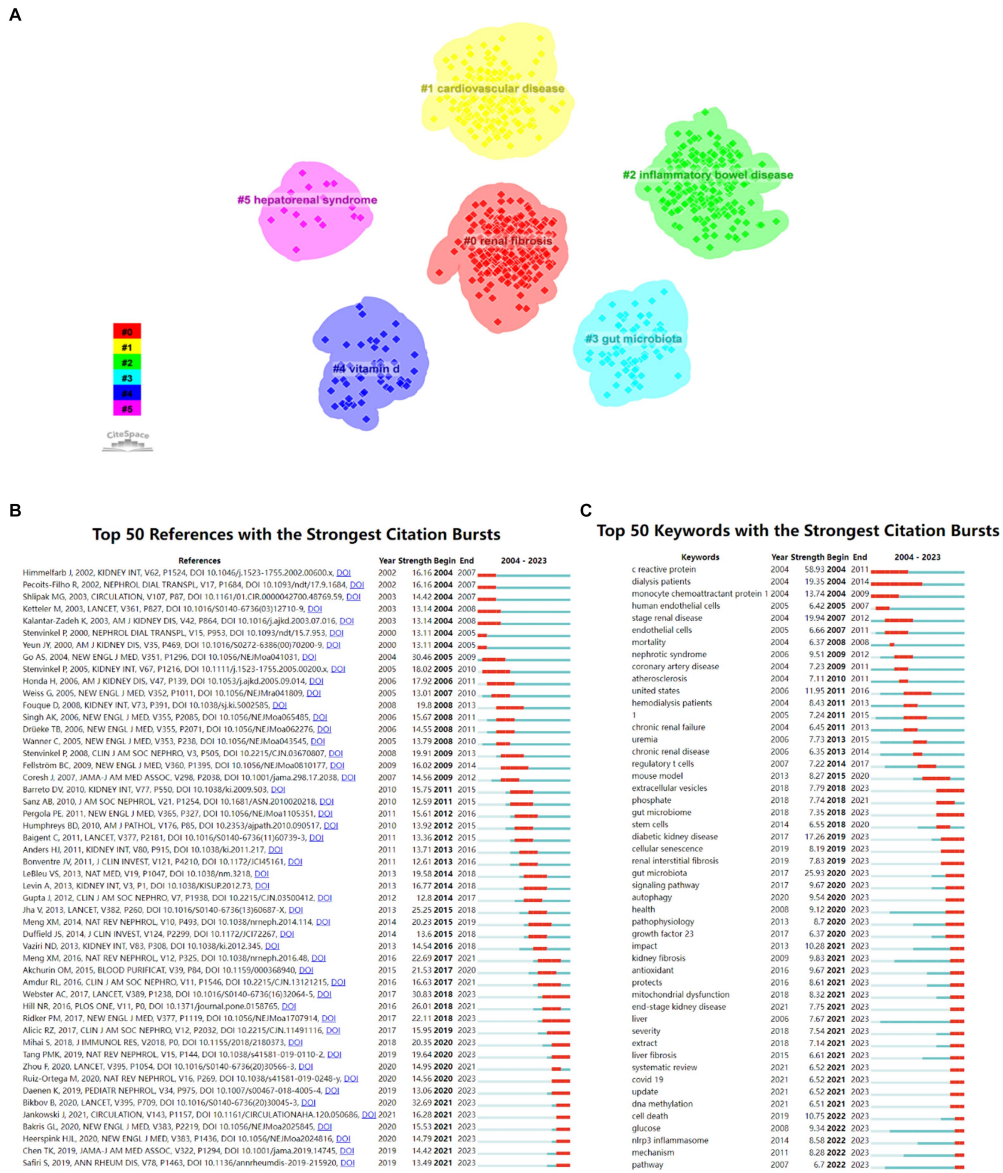
Research hotspots (**A**, keyword clustering diagram; **B**, cited literature emergence diagram; **C**, keyword emergence diagram).

By analyzing key words, we can quickly understand the situation and development direction of a field. According to the co-occurrence of keywords in VOSviewer, the most popular keywords were oxidative stress (1206), followed by expression (942), disease (907) and mortality (788; Table 7; Figures 8A,B). We removed useless keywords and constructed a network containing 186 keywords that appeared at least 78 times. It turned out a total of four different clusters. Group 1 (red) has 77 keywords, including expression, oxidative stress, activation, acute kidney injury, fibrosis, diabetic nephropathy, mesenchymal cells, nephropathy, mice, antioxidant, dysfunction, induction, lupus nephritis, liver, biomarker, nitric oxide synthase, macrophages, apoptosis, induction, damage, model, pathway. Group 2 (green) has 56 keywords, including tnf-alpha, C-reactive protein, mortality, pathogenesis, stage renal disease, obesity, smooth muscle cells, all cause, malnutrition, diabetes, association, cytokines, insulin resistance, atherosclerosis, vascular calibration, plasma, serum, protein, uremia, calcium, association, mortality, diabetes, malnutrition. The third group contains 51 keywords (blue), including risk, heart failure, therapy, prevalence, iron, management, efficiency, infection, quality of life, blood pressure, hypertension, health, outcomes, sepsis, impact, meta-analysis, cancer, deficiency, Crohn’s disease, COVID-19, children. Group four contains two keywords (yellow), including albuminuria and stress. We drew a volcano map with CiteSpace to visualize the changes of research hotspots keywords over time (Figures 8C, 9A).

**Table 7:** Top 20 high-frequency keywords.

Rank	Keyword	Counts	Rank	Keyword	Counts
1	oxidative stress	1,206	11	atherosclerosis	518
2	expression	942	12	injury	499
3	disease	907	13	association	482
4	mortality	788	14	acute kidney injury	437
5	risk	746	15	stage renal-disease	434
6	c-reactive protein	647	16	kidney	421
7	activation	639	17	mechanisms	401
8	cardiovascular-disease	567	18	NF-kappa-b	399
9	hemodialysis	528	19	hemodialysis-patients	395
10	fibrosis	522	20	progression	391

#### 3.7.4 Analysis of keywords with citation burst

The 50 most reliable citations in the field of the role of inflammation in CKD were obtained through CiteSpace. Among the 50 references, 43 were published from 2004 to 2023, indicating that they have been frequently cited in the past 20 years. Importantly, 13 of these papers are currently at the peak of Citation (Figure 9B), which suggests that the subject of the role of inflammation in CKD will remain a hot topic in the future. Among the 924 strongest burst keywords in this field, the 50 keywords with the strongest mutations (Figure 9C), which represent the current research hotspots in this field and the possible research direction in the future.

## 4 Discussion

This article aims to explore the relationship between inflammation and chronic kidney disease through bibliometric analysis, as well as to identify current hotspots and trends in research development. The number of publications in this field has been steadily increasing since 2007, with a sharp rise in 2013 and reaching its peak in 2022, indicating a growing interest in this area of research. The top three authors in terms of publication output are Stenvinkel, Peter (87, 0.936%), Lindholm, Bengt (59, 0.635%), Ortiz, Alberto (59, 0.635%), highlighting their significant contributions to this field. Co-citation network analysis reveals that an article titled “Chronic Kidney Disease” in The Lancet journal (IF = 168.9) has the highest citation frequency, indicating its significant impact and influence in this field.This article highlights the significant role of inflammatory factors in the development of numerous chronic kidney diseases (22). The common pathological manifestations of CKD include renal fibrosis, characterized by glomerulosclerosis, tubular atrophy, and interstitial fibrosis (23). Glomerulosclerosis is primarily caused by endothelial injury, dysfunction, proliferation of smooth muscle cells and mesangial cells, and podocyte damage within the glomerular basement membrane. However, the initial process is initiated by inflammation of the glomerulus due to the activation of endothelial cells, where inflammatory cells (including macrophages and foam cells) activate mesangial cell proliferation (24). Transforming growth factor β1 and other growth factors (including platelet-derived growth factor, fibroblast growth factor, tumor necrosis factor, and interferon-γ) stimulate mesangial cells to revert to mesangial progenitor cells (immature mesangial cells). These mesangial progenitor cells can produce excessive extracellular matrix, leading to mesangial expansion, early signs of glomerular fibrosis, and ultimately stretching of podocytes. This stretching exposes the area of the glomerular basement membrane to Bowman’s capsule, causing adhesion and resulting in glomerulosclerosis. Moreover, tubular atrophy is also associated with inflammatory products (25). The close relationship between tubular atrophy, interstitial fibrosis, scar formation, glomerular filtration rate, and proteinuria is well established. The abnormal filtration of urinary proteins, including complement, cytokines, and albumin, stimulates tubular epithelial cells, resulting in the synthesis of inflammatory products such as reactive oxygen species and chemokines. These inflammatory products are transported to the renal interstitium through mediators and interact with interstitial myofibroblasts, ultimately leading to tubular atrophy. Furthermore, mounting evidence suggests that inflammation, oxidative stress, uremia, and hyperhomocysteinemia may induce epigenetic changes that mediate fibrosis and play a significant role in the progression of chronic kidney disease (26).

After analyzing the literature, it is evident that the research trends and hotspots in this field primarily focus on the following aspects: (1) Inflammatory biomarkers related to chronic kidney disease and their roles. Research results indicate that persistent low-grade inflammation is one of the harmful pathways leading to increased cardiovascular morbidity and mortality in end-stage renal disease patients. Studies also suggest that inflammation is a risk factor for chronic kidney disease. Therefore, detecting the levels of inflammatory factors in CKD patients can provide a direct understanding of the extent of renal inflammation. Currently, commonly used biomarkers for microinflammation status in domestic and foreign research include CRP, hs-CRP, IL, TNF-α, etc. CRP is an acute-phase reactant protein produced by the liver under the stimulation of IL-6, IL-1β, TNF-α, etc., which can reflect the activity of inflammation in the body (27). The level of CRP in CKD patients is significantly elevated, and the baseline level of CRP is closely related to the risk of developing end-stage renal disease or death in patients (28). IL is a type of cytokine widely distributed in various cells, which can activate and regulate the immune system and mediate the body’s inflammatory response. However, in CKD patients, the imbalance of Th1/Th2 balance leads to abnormal expression of cytokines (IL-1, IL-4, IL-6, IL-10, etc.), which has a certain impact on the progression and prognosis of the disease. Studies have found that the elevation of IL-4 and IL-10 levels in CKD patients is closely related to the loss of renal function. IL-6 can mediate the apoptosis of renal tubular epithelial cells through signaling pathways and is an independent risk factor for poor prognosis in CKD patients. Elevated levels of interleukin-1 (IL-1), interleukin-6 (IL-IL6), and other inflammatory markers in patients with CKD activate the inflammatory cascade, exacerbating renal damage and promoting renal fibrosis (29–31). This suggests that monitoring the trend of these indicators in clinical practice can aid in evaluating the renal function of CKD patients. Additionally, studies have demonstrated that the interaction between soluble CD14 levels (a pattern recognition receptor that can stimulate pro-inflammatory signaling in response to endotoxins) and circulating IL-6 can increase the risk of mortality in uremic patients undergoing hemodialysis (32). Based on these research findings, preventive measures can effectively reduce inflammation, infection, and endotoxemia. Tumor necrosis factor-alpha (TNF-α) is a well-established classical inflammatory marker that plays a crucial role in the body’s inflammatory and immune responses (33). In CKD, studies have shown that neutrophil-to-lymphocyte ratio (NLR), platelet-to-lymphocyte ratio (PLR), CRP, TNF-α, and IL-6 are positively correlated and change in the same direction, reflecting the degree of inflammation in the body and the renal function status during the disease process, and are associated with poor prognosis. In recent years, there has been a growing interest in novel inflammatory markers in the field of nephrology research (34). These markers include the neutrophil-to-lymphocyte ratio (NLR), platelet-to-lymphocyte ratio (PLR), and fibrinogen-to-albumin ratio (FAR). Among these markers, FAR is calculated as the ratio of plasma fibrinogen to serum albumin, and it has gained increasing attention as a novel inflammatory marker. Studies have demonstrated that elevated FAR levels are an independent risk factor for the progression of kidney damage in patients with CKD. Compared to more commonly studied clinical markers of microinflammation, FAR has shown superior predictive value for disease progression (35). (2) Treatment of chronic kidney disease guided by inflammatory markers (36). Due to the significant role of inflammation in the development of chronic kidney disease, inflammatory markers have become the primary targets of novel treatments, including specific anti-inflammatory and antioxidant therapies to regulate inflammation. Specific anti-inflammatory therapies include cholecalciferol supplementation, which has been shown to decrease levels of CRP, IL-6, IL-8, and TNF (37). Additionally, the immunomodulatory effect of ARB is mainly related to the inhibition of pro-inflammatory cytokine secretion, the reduction of adhesion molecule expression, and the normalization of CRP concentration in plasma (38). Furthermore, allopurinol treatment in patients with chronic kidney disease can not only reduce CRP levels but also lower cardiovascular risk and delay the progression of kidney disease (39). Antioxidant therapy can modulate inflammation (40). Research has demonstrated that supplementation with γ-tocopherol and docosahexaenoic acid can reduce levels of IL-6 (41). Additionally, a randomized controlled trial has shown that short-term oral administration of N-acetylcysteine (NAC) can also reduce IL-6 levels (42). Methyl bardoxolone is a current research focus, which primarily inhibits pro-inflammatory transcription factors (such as NF-kB and STAT3) by activating the transcription factor Nrf2. Studies have indicated that it can not only prevent the progression of kidney disease but also treat uremic protein-energy wasting and cardiovascular disease (43).

## 5 Conclusion

In conclusion, the discovery of inflammatory markers in chronic kidney disease and the development of treatment methods for inflammation and oxidative stress have been regarded as research hotspots. Intervening in inflammatory factors, developing new prevention strategies, and treatment methods to reduce the incidence and mortality of chronic kidney disease remain the direction of future research.

## Data Availability

The original contributions presented in the study are included in the article/Supplementary material, further inquiries can be directed to the corresponding author.

## References

[1] Inker LA Astor BC Fox CH Isakova T Lash JP Peralta CA . KDOQI US commentary on the 2012 KDIGO clinical practice guideline for the evaluation and management of CKD. Am J Kidney Dis. (2014) 63:713–35. doi: 10.1053/j.ajkd.2014.01.416, PMID: 24647050

[2] Gu X Yang H Sheng X Ko YA Qiu C Park J . Kidney disease genetic risk variants alter lysosomal beta-mannosidase (MANBA) expression and disease severity. Sci Transl Med. (2021) 13:eaaz1458. doi: 10.1126/scitranslmed.aaz1458 33441424 PMC8627675

[3] Collaboration, G. C. K. D . Global, regional, and national burden of chronic kidney disease, 1990-2017: a systematic analysis for the global burden of disease study 2017. Lancet. (2020) 395:709–33. doi: 10.1016/S0140-6736(20)30045-3, PMID: 32061315 PMC7049905

[4] Chen TK Knicely DH Grams ME. Chronic kidney disease diagnosis and management: a review. JAMA. (2019) 322:1294–304. doi: 10.1001/jama.2019.14745, PMID: 31573641 PMC7015670

[5] Miyamoto T Carrero JJ Stenvinkel P. Inflammation as a risk factor and target for therapy in chronic kidney disease. Current Opinion in Nephrology & Hypertension. (2011) 20:662–8. doi: 10.1097/MNH.0b013e32834ad504, PMID: 21825982

[6] Wei J Zhang J Wang L Cha BJ Jiang S Liu R. A new low-nephron CKD model with hypertension, progressive decline of renal function, and enhanced inflammation in C57BL/6 mice. Am J Physiol Ren Physiol. (2018) 314:F1008–19. doi: 10.1152/ajprenal.00574.2017, PMID: 29412703 PMC6031904

[7] van Oosten M Logtenberg S Leegte M Bilo H Mohnen SM Hakkaart-van Roijen L . Age-related difference in health care use and costs of patients with chronic kidney disease and matched controls: analysis of Dutch health care claims data. Nephrol Dial Transplant. (2020) 35:2138–46. doi: 10.1093/ndt/gfz146, PMID: 31598728 PMC7716809

[8] Kadatane SP Satariano M Massey M Mongan K Raina R. The role of inflammation in CKD. Cells. (2023) 12:1581. doi: 10.3390/cells12121581, PMID: 37371050 PMC10296717

[9] Yoshida T Yamashita M Horimai C Hayashi M. Smooth muscle-selective nuclear factor-κB inhibition reduces phosphate-induced arterial medial calcification in mice with chronic kidney disease. J Am Heart Assoc. (2017) 6:1147–1154. doi: 10.1161/JAHA.117.007248, PMID: 29146611 PMC5721793

[10] Speer T Dimmeler S Schunk SJ Fliser D Ridker PM. Targeting innate immunity-driven inflammation in CKD and cardiovascular disease. Nat Rev Nephrol. (2022) 18:762–78. doi: 10.1038/s41581-022-00621-9, PMID: 36064794

[11] Graterol Torres F Molina M Soler-Majoral J Romero-González G Rodríguez Chitiva N Troya-Saborido M . Evolving concepts on inflammatory biomarkers and malnutrition in chronic kidney disease. Nutrients. (2022) 14:4297. doi: 10.3390/nu14204297, PMID: 36296981 PMC9611115

[12] Ueda N Takasawa K. Impact of inflammation on ferritin, Hepcidin and the Management of Iron Deficiency Anemia in chronic kidney disease. Nutrients. (2018) 10:1173. doi: 10.3390/nu10091173, PMID: 30150549 PMC6163440

[13] Mazzaferro S De Martini N Rotondi S Tartaglione L Ureña-Torres P Bover J . Bone, inflammation and chronic kidney disease. Clin Chim Acta. (2020) 506:236–40. doi: 10.1016/j.cca.2020.03.040, PMID: 32275989

[14] Kaysen GA . The microinflammatory state in uremia: causes and potential consequences. J Am Soc Nephrol. (2001) 12:1549–57. doi: 10.1681/ASN.V1271549, PMID: 11423586

[15] Stenvinkel P Chertow GM Devarajan P Levin A Andreoli SP Bangalore S . Chronic inflammation in chronic kidney disease progression: role of Nrf2. Kidney Int Rep. (2021) 6:1775–87. doi: 10.1016/j.ekir.2021.04.023, PMID: 34307974 PMC8258499

[16] Donthu N Kumar S Mukherjee D Pandey N Lim WM. How to conduct a bibliometric analysis: an overview and guidelines. J Bus Res. (2021) 133:285–96. doi: 10.1016/j.jbusres.2021.04.070, PMID: 39149772

[17] Ai S Li Y Zheng H Wang Z Liu W Tao J . Global research trends and hot spots on autophagy and kidney diseases: a bibliometric analysis from 2000 to 2022. Front Pharmacol. (2023) 14:1275792. doi: 10.3389/fphar.2023.1275792, PMID: 38099142 PMC10719858

[18] Sun HL Bai W Li XH Huang H Cui XL Cheung T . Schizophrenia and inflammation research: a bibliometric analysis. Front Immunol. (2022) 13:907851. doi: 10.3389/fimmu.2022.907851, PMID: 35757702 PMC9219580

[19] Lai PH Wang TH Zhang NY Wu KC Yao CJ Lin CJ. Changes of blood-brain-barrier function and transfer of amyloid beta in rats with collagen-induced arthritis. J Neuroinflammation. (2021) 18:35. doi: 10.1186/s12974-021-02086-2, PMID: 33516259 PMC7847579

[20] van Eck NJ Waltman L. Software survey: VOSviewer, a computer program for bibliometric mapping. Scientometrics. (2010) 84:523–38. doi: 10.1007/s11192-009-0146-3, PMID: 20585380 PMC2883932

[21] Synnestvedt MB Chen C Holmes JH. CiteSpace II: visualization and knowledge discovery in bibliographic databases. AMIA: Annual Symposium proceedings AMIA Symposium. (2005) 2005:724–8. PMID: 16779135 PMC1560567

[22] Webster AC Nagler EV Morton RL Masson P. Chronic kidney disease. Lancet. (2017) 389:1238–52. doi: 10.1016/S0140-6736(16)32064-5, PMID: 27887750

[23] Song MF Yi ZW Zhu XJ Qu XL Wang C Zhang ZQ . Statistical prediction in pathological types of chronic kidney disease. Chin Med J. (2018) 131:2741–2. doi: 10.4103/0366-6999.245273, PMID: 30425201 PMC6247582

[24] Alkhatib L Velez Diaz LA Varma S Chowdhary A Bapat P Pan H . Lifestyle modifications and nutritional and therapeutic interventions in delaying the progression of chronic kidney disease: a review. Cureus. (2023) 15:e34572. doi: 10.7759/cureus.34572, PMID: 36874334 PMC9981552

[25] Sarakpi T Mesic A Speer T. Leukocyte-endothelial interaction in CKD. Clin Kidney J. (2023) 16:1845–60. doi: 10.1093/ckj/sfad135, PMID: 37915921 PMC10616504

[26] Luyckx VA Cherney D Bello AK. Preventing CKD in developed countries. Kidney Int Rep. (2020) 5:263–77. doi: 10.1016/j.ekir.2019.12.003, PMID: 32154448 PMC7056854

[27] Rizo-Téllez SA Sekheri M Filep JG. C-reactive protein: a target for therapy to reduce inflammation. Front Immunol. (2023) 14:1237729. doi: 10.3389/fimmu.2023.1237729 37564640 PMC10410079

[28] Li J Chen J Lan HY Tang Y. Role of C-reactive protein in kidney diseases. Kidney Dis (Basel, Switzerland). (2023) 9:73–81. doi: 10.1159/000528693, PMID: 37065607 PMC10090978

[29] Nowak KL Chonchol M Ikizler TA Farmer-Bailey H Salas N Chaudhry R . IL-1 inhibition and vascular function in CKD. J Am Soc Nephrol. (2017) 28:971–80. doi: 10.1681/ASN.2016040453, PMID: 27647856 PMC5328163

[30] Wei W Zhao Y Zhang Y Jin H Shou S. The role of IL-10 in kidney disease. Int Immunopharmacol. (2022) 108:108917. doi: 10.1016/j.intimp.2022.108917, PMID: 35729842

[31] Zoccali C Mallamaci F. Innate immunity system in patients with cardiovascular and kidney disease. Circ Res. (2023) 132:915–32. doi: 10.1161/CIRCRESAHA.122.321749, PMID: 37053283

[32] Rogacev KS Seiler S Zawada AM Reichart B Herath E Roth D . CD14++CD16+ monocytes and cardiovascular outcome in patients with chronic kidney disease. Eur Heart J. (2011) 32:84–92. doi: 10.1093/eurheartj/ehq371, PMID: 20943670

[33] Zelová H Hošek J. TNF-α signalling and inflammation: interactions between old acquaintances. Inflamm Res. (2013) 62:641–51. doi: 10.1007/s00011-013-0633-0, PMID: 23685857

[34] Huang P Mai Y Zhao J Yi Y Wen Y. Association of systemic immune-inflammation index and systemic inflammation response index with chronic kidney disease: observational study of 40,937 adults. Inflamm Res. (2024) 73:655–67. doi: 10.1007/s00011-024-01861-0, PMID: 38489048

[35] Zhu Y Tao S Zhang D Xiao J Wang X Yuan L . Association between fibrinogen/albumin ratio and severity of coronary artery calcification in patients with chronic kidney disease: a retrospective study. PeerJ. (2022) 10:e13550. doi: 10.7717/peerj.13550, PMID: 35694387 PMC9179587

[36] Thomas JM Huuskes BM Sobey CG Drummond GR Vinh A. The IL-18/IL-18R1 signalling axis: diagnostic and therapeutic potential in hypertension and chronic kidney disease. Pharmacol Ther. (2022) 239:108191. doi: 10.1016/j.pharmthera.2022.108191, PMID: 35461924

[37] Meireles MS Kamimura MA Dalboni MA Giffoni de Carvalho JT Aoike DT Cuppari L. Effect of cholecalciferol on vitamin D-regulatory proteins in monocytes and on inflammatory markers in dialysis patients: a randomized controlled trial. Clinical Nutrit (Edinburgh, Scotland). (2016) 35:1251–8. doi: 10.1016/j.clnu.2016.04.014, PMID: 27161894

[38] Bryniarski P Nazimek K Marcinkiewicz J. Immunomodulatory activity of the Most commonly used antihypertensive drugs-angiotensin converting enzyme inhibitors and angiotensin II receptor blockers. Int J Mol Sci. (2022) 23:1772. doi: 10.3390/ijms23031772, PMID: 35163696 PMC8836033

[39] Usalan Ö Şahin AZ Özdemir O Cingöz M Usalan C. Effect of allopurinol drug use on GFR and proteinuria in patients with renal transplant recipients (ADOPTR study). Transpl Immunol. (2022) 72:101560. doi: 10.1016/j.trim.2022.101560, PMID: 35245661

[40] Jun M Venkataraman V Razavian M Cooper B Zoungas S Ninomiya T . Antioxidants for chronic kidney disease. Cochrane Database Syst Rev. (2012) 10:CD008176. doi: 10.1002/14651858.CD008176.pub2, PMID: 23076940 PMC8941641

[41] Shing CM Fassett RG Peake JM Coombes JS. Effect of tocopherol on atherosclerosis, vascular function, and inflammation in apolipoprotein E knockout mice with subtotal nephrectomy. Cardiovasc Ther. (2014) 32:270–5. doi: 10.1111/1755-5922.12096, PMID: 25307205

[42] Schepers E Barreto DV Liabeuf S Glorieux G Eloot S Barreto FC . Symmetric dimethylarginine as a proinflammatory agent in chronic kidney disease. Clin J Am Soc Nephrol. (2011) 6:2374–83. doi: 10.2215/CJN.01720211 21817129 PMC3359555

[43] Pergola PE Raskin P Toto RD Meyer CJ Huff JW Grossman EB . Bardoxolone methyl and kidney function in CKD with type 2 diabetes. N Engl J Med. (2011) 365:327–36. doi: 10.1056/NEJMoa1105351, PMID: 21699484

