# Supplementary material for: Chronic kidney diseases and inflammation research: a bibliometric analysis
Source: Front Med (Lausanne). 2024 Sep 20;11:1388665. doi: 10.3389/fmed.2024.1388665 (PMC11449749; doi:10.3389/fmed.2024.1388665)
Supplement: Supplementary file 1 [file Image_1.pdf]

PRISMA 2020 flow diagram for new systematic reviews which included searches of databases and registers only

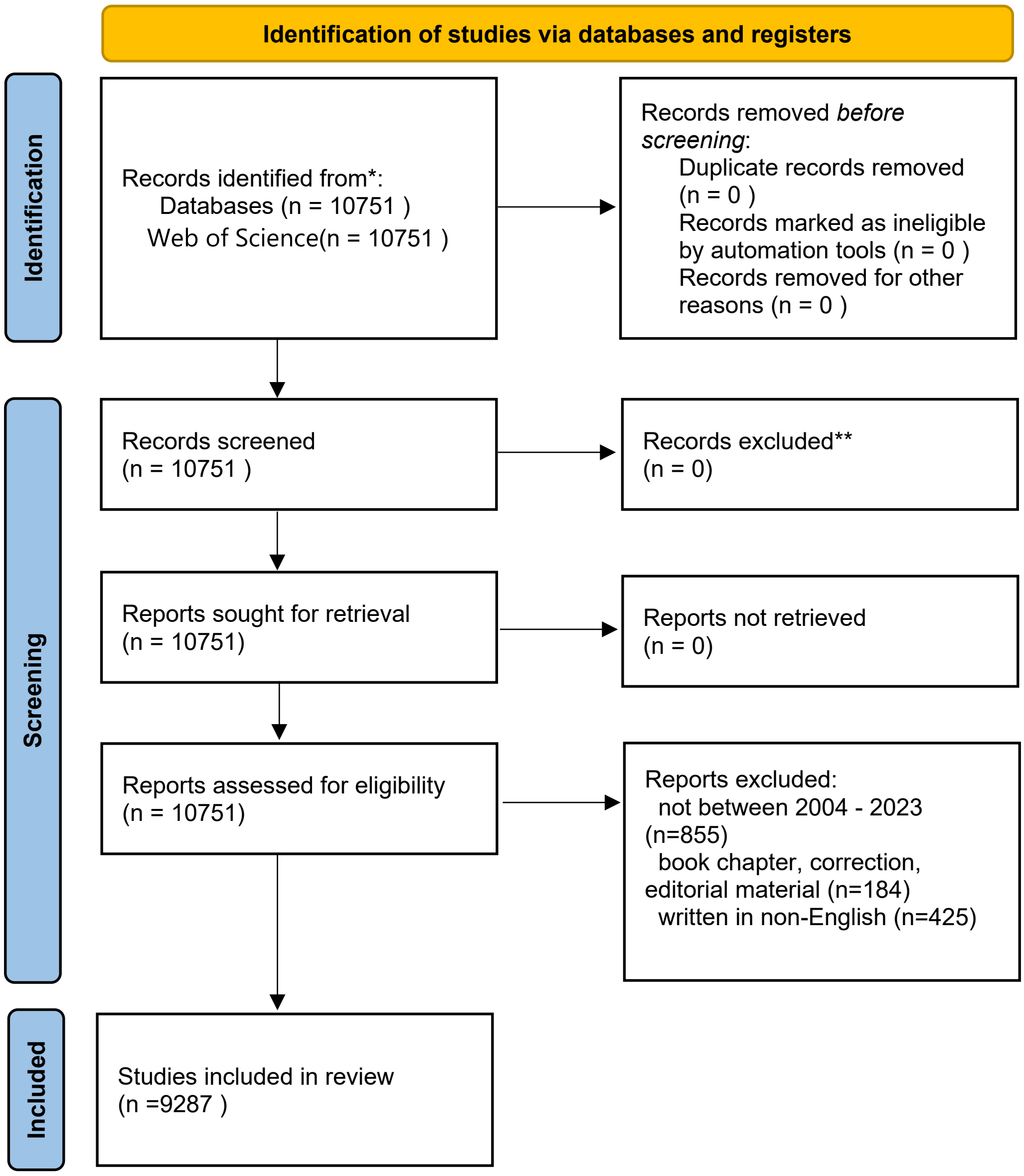

*Consider, if feasible to do so, reporting the number of records identified from each database or register searched (rather than the total number across all databases/registers).

**If automation tools were used, indicate how many records were excluded by a human and how many were excluded by automation tools.

Source: Page MJ, et al. BMJ 2021;372:n71. doi: 10.1136/bmj.n71.

This work is licensed under CC BY 4.0. To view a copy of this license, visit https://creativecommons.org/licenses/by/4.0/
